# Prenatal auditory learning in avian vocal learners and non-learners

**DOI:** 10.1098/rstb.2020.0247

**Published:** 2021-10-25

**Authors:** Diane Colombelli-Négrel, Mark E. Hauber, Christine Evans, Andrew C. Katsis, Lyanne Brouwer, Nicolas M. Adreani, Sonia Kleindorfer

**Affiliations:** ^1^ College of Science and Engineering, Flinders University, Adelaide 5001, Australia; ^2^ Department of Evolution, Ecology, and Behavior, School of Integrative Biology, University of Illinois at Urbana-Champaign, Urbana, IL 61801, USA; ^3^ Department of Animal Ecology & Physiology, Institute for Water and Wetland Research, Radboud University, Nijmegen, The Netherlands; ^4^ Department of Animal Ecology, Netherlands Institute of Ecology (NIOO-KNAW), Wageningen, The Netherlands; ^5^ Division of Ecology and Evolution, Research School of Biology, The Australian National University, Canberra, ACT 2601, Australia; ^6^ Faculty of Life Sciences, University of Vienna, Vienna, Austria

**Keywords:** *in ovo* learning, embryonic discrimination, playback

## Abstract

Understanding when learning begins is critical for identifying the factors that shape both the developmental course and the function of information acquisition. Until recently, sufficient development of the neural substrates for any sort of vocal learning to begin in songbirds was thought to be reached well after hatching. New research shows that embryonic gene activation and the outcome of vocal learning can be modulated by sound exposure *in ovo*. We tested whether avian embryos across lineages differ in their auditory response strength and sound learning *in ovo*, which we studied in vocal learning (Maluridae, Geospizidae) and vocal non-learning (Phasianidae, Spheniscidae) taxa. While measuring heart rate *in ovo*, we exposed embryos to (i) conspecific or heterospecific vocalizations, to determine their response strength, and (ii) conspecific vocalizations repeatedly, to quantify cardiac habituation, a form of non-associative learning. Response strength towards conspecific vocalizations was greater in two species with vocal production learning compared to two species without. Response patterns consistent with non-associative auditory learning occurred in all species. Our results demonstrate a capacity to perceive and learn to recognize sounds *in ovo*, as evidenced by habituation, even in species that were previously assumed to have little, if any, vocal production learning.

This article is part of the theme issue ‘Vocal learning in animals and humans’.

## 1. Introduction

Vocal learning [1] is generally defined as the capacity to produce vocalizations based on the imitation of external sounds (vocal production learning), but can extend to the capacity to associate a vocalization with an outcome (comprehension learning) or to produce an existing vocalization in a novel context (vocal usage learning) (reviewed in [2]). However, vocal learning is often presented as a binary behaviour whereby species are classified as either vocal learners or non-learners. To overcome this probably incorrect simplification, new research directions advocate for a multimodal vocal learning continuum based upon increasing complexity in species' vocal learning capacities (behavioural, neurobiological, molecular) [2–4]. Most so-called ‘vocal non-learners’ have been classified as non-learners owing to their phylogenetic affinities with other non-learners, or by omission, because we lack experimental tests of their discriminatory or learning capacity. Hence, there is a need for cross-species comparisons to fill these major gaps in knowledge before conclusions about vocal learning are made.

Species-specific vocalizations vary significantly across species; recognizing and producing the right vocalization can be critical to interact with conspecifics [5]. Such vocalizations elicit a stronger neural and behavioural response than non-specific sounds ([5], reviewed in [6]), even in acoustically naïve birds, and in the absence of prior conspecific stimulation [7,8]. Based on such findings, responses to species-specific vocalizations have often been assumed to be the result of ‘innate’ auditory predispositions that are at least partly genetically determined [7,9], despite evidence that vocal templates can be acquired well before hatching (e.g. [10–12]).

**Figure 1.**
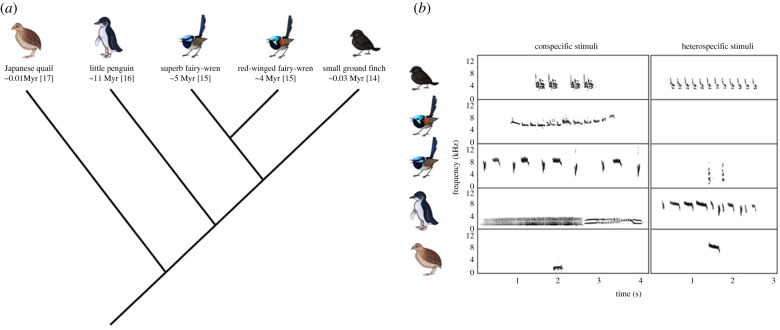
(*a*) Simplified phylogenetic tree of the five avian species used in this study and (*b*) spectrograms of the conspecific and heterospecific vocalizations used in this study. In (*a*), we present under each species name the time since divergence from the ancestral sister species in millions of years (Myr), as well as the relevant reference(s) in brackets. (Online version in colour.)

Here, we measured response strength and non-associative learning (habituation) of vocalizations in avian embryos with different characteristics (e.g. vocal learners versus non-learners; altricial and semi-altricial versus precocial; ancient versus recent lineages). We selected these two rudimentary cognitive dimensions because they are considered fundamental components of vocal learning, and yet there is no comparative information on their general occurrence (or absence) across avian taxa, nor extensive experimental tests of these capacities *in ovo*. The response was measured as a change in heart rate (HR). In birds and humans, a drop in embryonic HR has been shown to reflect physiological mechanisms for orientation and attention [10,13]. The study systems included five species from four avian families: Maluridae and Geospizidae (typically classified as vocal learners) and Phasianidae and Spheniscidae (typically classified as vocal non-learners) (figure 1). We first exposed embryos of four avian species to conspecific or heterospecific vocalizations and measured their auditory response strengths across 60 s. We then exposed embryos of five avian species to 180 s of conspecific vocalizations to measure their potential cardiac habituation response (defined as a decrease in response strength to repeated stimulation of the same stimulus type [18–20]). We predicted that the precocial embryos (Phasianidae; classified here also as a vocal non-learner) would exhibit larger physiological effects in both experiments because they are physiologically and morphologically more developed at the same comparative age *in ovo* than the semi-altricial (Spheniscidae; classified here also as vocal non-learners) and altricial embryos (oscine Maluridae and Geospizidae; both classified here also as vocal learners).

## 2. Methods

### (a) Study species

Between 2012 and 2019, we measured HR in embryos across five species: (i) the superb fairy-wren (*Malurus cyaneus*; 2012–2014, 2019); (ii) the red-winged fairy-wren (*Malurus elegans*; 2016)—both insectivorous passerines from southern Australia [21]; (iii) Darwin's small ground finch (*Geospiza fuliginosa*; 2016)—one of 17 Darwin's finch species on the Galápagos Islands [22]; (iv) the little penguin (*Eudyptula minor*; 2014, 2015, 2016)—a seabird endemic to southern Australia and New Zealand [23]; and (v) the Japanese quail (*Coturnix japonica domestica*; 2014)—a domesticated species derived from the Eurasian quail (*Coturnix coturnix*) [24]. Electronic supplementary material, table S1 presents the characteristics of each species and figure 1 their phylogenetic representation. We conducted all our experiments in the field on wild eggs, except for those using Japanese quail (see the electronic supplementary material), and measured HR using a digital egg monitor (Buddy™, Vetronic Services, UK) following published methods [10,25,26].

### (b) Experiment 1. Response strength

We exposed embryos to 60 s of pre-playback silence (baseline) followed by 60 s of playback and 60 s of silence (post-playback). The 60 s of playback included either (i) one conspecific or (ii) one heterospecific call or song from the same individual every 10 s. All embryos were tested once with one randomly chosen stimulus type (either conspecific or heterospecific). None of the conspecific vocalizations were from individuals known or related to the embryos. Electronic supplementary material, table S2 presents the list of vocalizations used for each species (see also figure 1). We tested 109 embryos across four species: superb fairy-wren (*n* = 33), small ground finch (*n* = 18), little penguin (*n* = 25) and Japanese quail (*n* = 33).

### (c) Experiment 2. Non-associative sound learning (habituation)

To measure their habituation response, we presented each embryo with 60 s of pre-playback silence (baseline), followed by three sets (referred to as H1, H2, H3) of 60 s of playback followed by 60 s of silence (electronic supplementary material, figure S1). Each 60 s of playback included different conspecific call or songs from the same individual (total 18 different conspecific calls or songs) repeated every 10 s (individuals differed between embryos). Habituation is defined as a decline in the frequency or magnitude of response with repeated stimulation or presentations (criterion 1), often followed by a lack of further shift or apparent response after a certain number of repetitions (criterion 2) [18–20]. As in most other studies (e.g. [10,27–29], we considered an individual to have habituated if criterion 1 was fulfilled (H3 < H1; criterion 1), but also checked if it maintained comparable HR with the preceding set (H2 = H3; criterion 2). The response during H1 was calculated as HR during H1 minus HR during baseline; the response during H2 was calculated as HR during H2 minus H1; the response during H3 was calculated as HR during H3 minus H2. We tested 138 embryos across five species: superb fairy-wren (*n* = 56), red-winged fairy-wren (*n* = 32), small ground finch (*n* = 15), little penguin (*n* = 19) and Japanese quail (*n* = 16). In the electronic supplementary material, we present the findings of the dishabituation response to support the claim that we measured a habituation response (electronic supplementary material table S3 and figure S2).

### (d) Statistical analyses

The statistical analyses were performed using the packages ‘lme4’ [30] and ‘arm’ [31] in R-3.3.3 [32] in a pseudo-Bayesian framework with non-informative priors. We used linear mixed-effect models with a Gaussian error distribution for all analyses and performed visual inspections of the residuals to assess the models' fit. The posterior distributions of the model parameters were obtained using the function ‘sim’. We carried out 10 000 simulations to extract the mean estimates and 95% credible intervals [33]. Statistical support was inferred from the posterior distribution of each parameter [34]. We considered an effect to be statistically meaningful when the posterior probability of the mean difference between compared estimates (termed ‘*p(dif)*’) was higher than 95% [34]. See details of the models in the electronic supplementary material.

## 3. Results

### (a) Experiment 1. Response strength

**Figure 2.**
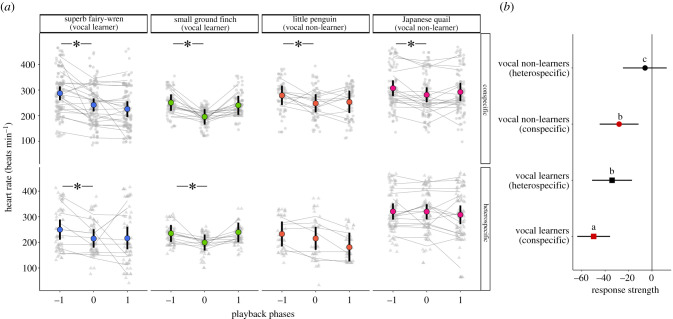
Experiment 1: response strength (*n* = 109). (*a*) HR (beats min^−1^) of the four species tested in our study during the pre-playback (-1), playback (0) and post-playback (1) phases in response to two stimuli: conspecific and heterospecific. Grey shapes represent the raw data (every egg has six repeated measures within each phase) and lines the mean change in HR for each egg. Coloured shapes represent the model estimates and the black bars the 95% credible intervals (CrI). (*b*) Response strength (*posteriori* distribution of playback HR minus pre-playback HR; beats min^−1^) of vocal learners (squares) and non-learners (circles) from the pre-playback to the playback phase in response to two stimuli: conspecific (red) and heterospecific (black). Circles represent the mean estimated difference between the pre-playback and playback derived from the posterior distribution of the model estimates and horizontal bars the 95% CrI. Asterisks/letters indicate statistical difference (i.e. *p(dif)* > 95%), with ‘a’ different from ‘b’ and ‘b’ different from ‘c’. (Online version in colour.)

There was no statistical difference in baseline HR (pre-playback) between the two playback groups (conspecific versus heterospecific) in any of the four species tested (*p(dif)* < 49.31% for every species; electronic supplementary material, table S4; figure 2*a*). Both vocal learner and non-learner species responded to conspecific vocalizations (*p(dif)*_Vocal learners_ = 99.99%; *p(dif)*_Vocal non-learners_ = 99.96%; electronic supplementary material, table S5; figure 2*b*) and the response strength was considerably larger in the vocal learners compared to non-learners (*p(dif)* = 97.59%; *electronic supplementary material,* table S5; figure 2*b*). Only vocal learners responded towards heterospecific stimuli (*p(dif)*_Vocal learners_ = 99.99%; *p(dif)*_Vocal non-learners_ = 73.65%; electronic supplementary material, table S5; figure 2*b*), and this effect was smaller when compared to their responses towards conspecific stimuli (*p(dif)* = 96.35%; electronic supplementary material, table S5; figure 2*b*).

### (b) Experiment 2. Non-associative sound learning (habituation)

**Figure 3.**
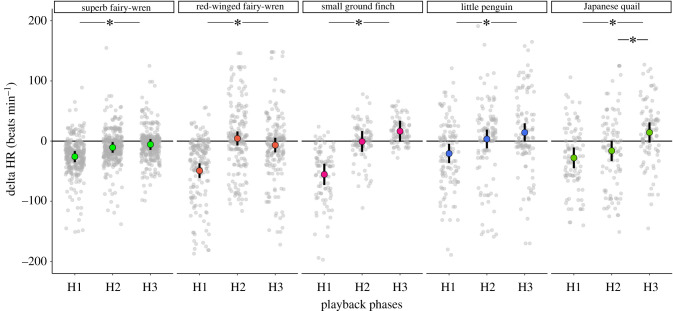
Experiment 2: non-associative sound learning (habituation) (*n* = 138). Delta HR (average change in HR during each phase relative to HR in the preceding playback phase; beats min^−1^) of the five species tested in our study exposed to three sets (H1, H2 and H3) of six different vocalizations of the same conspecific. Grey dots represent the raw data (each egg has multiple measures within each set of stimuli). Coloured circles represent the model estimates and the black bars the 95% credible intervals. Asterisks indicate statistical difference (i.e. *p(dif)* > 95%). (Online version in colour.)

Embryos significantly lowered their HR in response to the first set of vocalizations from a conspecific (H1 < baseline) in all five of the species tested (*p(dif)* > 99.9% for every species; electronic supplementary material, table S6; figure 3). Embryos from all species showed a decrease in delta HR between H1 and H3 (i.e. lower H3 values calculated as H3 minus H2 compared with stronger H1 values calculated as H1 minus baseline, indicating a habituation response (criterion 1); electronic supplementary material, table S6; figure 3). Strong support for this pattern was found in all five species (superb fairy-wren: *p(dif)* > 99.99%; red-winged fairy-wren: *p(dif)* *=* 99.99%; small ground finch: *p(dif)* *>* 99.99%; little penguin: *p(dif)* *>* 99.99%; Japanese quail: *p(dif)* > 99.99%; electronic supplementary material, table S6; figure 3). Superb fairy-wrens, red-winged fairy-wrens, small ground finches and little penguins maintained comparable delta HR between H2 and H3 (superb fairy-wren: *p(dif)* *=* 74.30%; red-winged fairy-wren: *p(dif)* *=* 86.13%; small ground finch: *p(dif)* *=* 86.63%; little penguin: *p(dif)* *=* 78.25%) electronic supplementary material, table S6; figure 3) whereas Japanese quail continued to respond to the stimuli and had not yet fully habituated (criterion 2). Our dishabituation tests described in the electronic supplementary material also show an overall dishabituation response when the embryos were exposed to novel stimuli (electronic supplementary material, table S3 and figure S2).

## 4. Discussion

Vocal production learning is only believed to occur in approximately eight lineages of birds and mammals, including humans (reviewed in [2]). To understand the complexity of vocal learning, it is, therefore, important to determine ‘which species are capable of which forms of vocal learning’ [1, p. 1]. With a better understanding of the ontogeny of response strength to sound, we can test predictions about the potential for early-life experience to influence neural pathways associated with vocal learning. Here, we showed that embryos of a diverse group of avian species with different characteristics responded more strongly to conspecific vocalizations than to heterospecific vocalizations, and that overall response strength towards conspecific vocalizations was greater in vocal learners than non-learners. By contrast, and contrary to our prediction, we found a similar pattern of habituation response in all species when embryos were exposed to repeated stimuli. The findings of this study suggest that the capacity to perceive and habituate to sound *in ovo* in developing birds may be more widespread taxonomically than previously considered and also support the idea that vocal perception learning is not a binary behaviour.

Precocial embryos (Japanese quail; classified as a vocal non-learner) hatch at a much later relative stage of development compared to semi-altricial (little penguin; classified as a vocal non-learner) or altricial (oscine Maluridae and Geospizidae; classified as vocal learners) offspring*.* Despite this, we found a significant difference in response strengths between vocal learners and non-learners, which aligns with the idea that limited (‘the ability to fine-tune acoustic features of species-specific vocalizations’) or complex (‘the need to hear a sound to form a learned auditory template before the animal can develop a vocalization that matches the template’) vocal learning may involve different neural recognition templates [1, p. 1]. Many studies claim to show genetic predispositions for species-typical vocalizations that are modified by experience [7,9]. Here, the altricial vocal learners appeared to have more fine-tuned response strengths at a much earlier stage of development compared with the precocial and semi-altricial vocal non-learners. This may be owing to our selection of heterospecific stimuli (all songbird species), which may not be ecologically relevant to the non-learner species. Our results may also reflect physiological differences linked to developmental modes (precocial versus semi-altricial and altricial), and further testing using altricial vocal non-learner species would help to clarify the effects of various component variables. However, the pattern that little penguin embryos (semi-altricial; vocal non-learner) performed similarly to the Japanese quail embryos (precocial; vocal non-learner) tends to suggest that the differences may be owing to differences in vocal production learning. It may also be that altricial vocal learning species have different timing to acquire conspecific recognition templates compared with precocial and semi-altricial vocal non-learning species, in which vocal production learning is expected to be limited. Additional studies comparing species are clearly needed to differentiate between these possibilities.

Our finding of shared response patterns to habituation stimuli leads us to conclude that, in general, avian embryos have the capacity to perceive sound and learn a response to sound *in ovo*. This implies that prenatal auditory experience may guide an individual's attention to cues experienced pre-hatch (and early post-hatch) and may result in different patterns of attention towards familiar and unfamiliar stimuli (see also [35]). The role of carry-over effects from previous habituation stimuli still needs to be tested, as well as the role of enhanced response strength to either familiar or unfamiliar stimuli following prior habituation response. At the mechanistic level, prenatal sound experience may result in earlier onset of gene expression and neural pathway establishment than previously imagined [11]. During subsequent ontogenetic stages, the diversity of prenatal sound experience may influence an individual's proximity to or preference for other individuals with a narrow or broad vocal repertoire, including their selection of vocal tutors [25,36], neighbours or neighbourhoods.

In conclusion, this study offers new avenues to look at early-life effects on the ontogeny of auditory learning. We showed an early onset of auditory recognition learning (i.e. vocal perception learning) in all families of avian embryos tested and found initial evidence for lineage differences in response strength to different categories of sound experienced *in ovo*. Future research could address changes in neural organization and gene expression pathways in embryos reared in sound-poor or -rich environments, identify how prenatal sound experience shapes phenotypic variation in vocalization characteristics, measure the effect of pre-hatch acoustical environment on individual attention to particular cues/signals/environments post-hatch, and quantify the strength of selection on adults to guide the prenatal sound experience of their offspring.

## Data accessibility

All data are available from the Dryad Digital Repository: https://doi.org/10.5061/dryad.v9s4mw6tt [37].
